# Fetomaternal outcome in preeclampsia and eclampsia with posterior reversible encephalopathy syndrome

**DOI:** 10.5339/qmj.2024.59

**Published:** 2024-11-11

**Authors:** Upma Saxena, Salimun Nisa, Yatish Agarwal, Abhishek Lachyan, S. K. Chandan, Sidarrth Prasad

**Affiliations:** 1 Department of Obstetrics and Gynecology, VMMC and Safdarjung Hospital, New Delhi, India *Email: drupmasaxena@gmail.com; 2 Department of Radiodiagnosis, VMMC and Safdarjung Hospital, New Delhi, India; 3 Department of Neurology, VMMC and Safdarjung Hospital, New Delhi, India; 4 Department of Neurology, University of Texas Southwestern Medical Center, Dallas, TX, USA

**Keywords:** Cesarean section, eclampsia, fetomaternal outcome, high-risk pregnancy, intubation, neurological symptoms, NICU stay, posterior reversible encephalopathy syndrome (PRES), prematurity, preeclampsia

## Abstract

**Introduction:**

Preeclampsia and eclampsia are hypertensive disorders of pregnancy associated with significant maternal and fetal morbidity and mortality. Posterior reversible encephalopathy syndrome (PRES) is a neurological complication observed in these conditions, yet its impact on fetomaternal outcomes remains underexplored. The aim of this study is to investigate the association between PRES and fetomaternal outcomes in women with preeclampsia and eclampsia.

**Methods:**

This prospective cohort study enrolled 64 singleton pregnant women beyond 20 weeks of gestation with preeclampsia and eclampsia having neurological symptoms. Participants underwent clinical evaluation and radiological assessment, including non-contrast computed tomography (NCCT) and magnetic resonance imaging (MRI). Maternal outcomes, including high dependency unit (HDU)/intensive care unit (ICU) stay, intubation, mode of delivery and maternal mortality. Fetal outcomes, i.e., stillbirth, prematurity, and neonatal intensive care unit (NICU) stay, were recorded. Statistical analysis was performed to compare outcomes between PRES and non-PRES groups.

**Results:**

The majority (92.18%) of participants were unscheduled and (56.2%) were primigravida. PRES was diagnosed in 62.5% of cases, predominantly associated with antepartum eclampsia (47.5%). Neurological symptoms such as headache (85.9%) and blurring of vision (68.8%) were common in PRES cases. Cesarean section rate was significantly higher in PRES group (75%), along with adverse outcomes including increased HDU/ICU stay, intubation, postpartum haemorrhage (PPH), abruption, maternal mortality, stillbirth, prematurity, fetal growth restriction (FGR), meconium-stained liquor (MSL), and NICU stay, which were observed significantly higher in women with PRES (*p* < 0.001) and low Appearance, Pulse, Grimace, Activity, and Respiration (APGAR) scores (*p* < 0.05). However, no significant association was observed between PRES and early neonatal death (ENND).

**Conclusion:**

PRES is associated with adverse fetomaternal outcomes in women with preeclampsia/eclampsia, including higher cesarean section rate and increased maternal and neonatal morbidity. Early recognition and management of PRES are crucial for improving outcomes in these high-risk pregnancies. Further research with larger sample sizes is warranted to validate these findings and explore potential interventions.

## 1. Introduction

Preeclampsia and eclampsia remain significant concerns in maternal healthcare, particularly in regions like India where their incidence is notable. Preeclampsia, a hypertensive disorder occurring in 8–10% of pregnancies, is associated with a spectrum of complications, contributing to maternal mortality rates ranging from 9% to 26% in low-income countries and 16% in high-income nations.^1^ Gestational hypertension, according to American College of Obstetricians and Gynecologists (ACOG) guidelines, is characterized by a blood pressure of 140 mmHg or higher systolic, or 90 mmHg or higher diastolic, on two separate occasions at least 4 hours apart after 20 weeks of pregnancy in women with previously normal blood pressure. Alternatively, if a patient’s systolic blood pressure exceeds 160 mmHg or diastolic blood pressure exceeds 110 mmHg, a diagnosis of gestational hypertension can be confirmed with a similar reading after a short interval to ensure timely antihypertensive treatment. Clinical symptoms typically become apparent when blood pressure exceeds 160/110 mmHg, potentially indicating end-organ damage.^2^

Preeclampsia, as defined by ACOG guidelines, involves meeting the hypertension criteria described above along with either a urine protein excretion of 300 mg or more in a 24-hour period or a protein/creatinine ratio of 0.3 or higher. If these methods are unavailable, a urine dipstick test can be used, with proteinuria indicated by a protein reading of at least 1+.^2^ Preeclampsia poses substantial risks to both maternal and fetal health. Various risk factors, including nulliparity, advanced maternal age, and comorbidities such as chronic hypertension and diabetes mellitus, contribute to the development of preeclampsia. The condition can progress to severe stages, marked by more profound hypertension and multi-organ involvement, warranting immediate medical intervention to mitigate adverse outcomes.^3,4^

Eclampsia, a convulsive manifestation of hypertensive disorders of pregnancy, represents one of the most severe complications, often culminating in significant morbidity and mortality. Neuroimaging manifestations of both preeclampsia and eclampsia frequently converge on posterior reversible encephalopathy syndrome (PRES), characterized by distinct clinicopathologic features, including headaches, visual disturbances, and seizures.^5,6^

The pathophysiology of PRES and its association with preeclampsia remain areas of ongoing research. Endothelial dysfunction is implicated in both conditions, suggesting a common underlying mechanism. Dysregulation of microRNAs (miRNAs) is proposed as a contributing factor, affecting placental development and vascular function. Radiological imaging, particularly magnetic resonance imaging (MRI), plays a pivotal role in diagnosing PRES, revealing characteristic lesions primarily in the parieto-occipital lobes. The prevalence of PRES in patients with preeclampsia ranges from 2% to 7%. This variability is due to differences in study populations, diagnostic criteria, and imaging techniques used to identify PRES.^7,8,9^ In contrast, the prevalence of PRES in patients with eclampsia is significantly higher, ranging from 20% to 40%. Eclampsia, characterized by seizures in the setting of preeclampsia, has a stronger association with the development of PRES.^8,9^

While PRES lesions are more prevalent in eclampsia, they also occur in a substantial proportion of severe preeclampsia cases, often correlating with neurological symptoms.^10,11^

Management strategies for PRES focus on addressing underlying causes, controlling blood pressure, and symptomatic management with antiepileptic and antihypertensive medications. However, there is currently no established antiepileptic regimen specific to PRES.^12,13^ This study aims to investigate the fetomaternal outcomes in women with preeclampsia and eclampsia presenting with neurological symptoms suggestive of PRES. By elucidating the impact of PRES on maternal and fetal health, we seek to enhance our understanding of these complex conditions and inform targeted interventions to improve outcomes for both mothers and infants.

## 2. Material and Methods

This prospective observational cohort study was conducted at Vardhman Mahavir Medical College and Safdarjung Hospital, New Delhi, in collaboration with the Departments of Obstetrics and Gynaecology, Radiodiagnosis, and Neurology. Ethical clearance was obtained from the Institutional Ethics Committee, and written informed consent was secured from all participants. Over a duration of 18 months i.e. August 1, 2022, and January 31, 2024, a sample size of 64 singleton pregnant women beyond 20 weeks of gestation, diagnosed with preeclampsia or eclampsia and presenting with neurological symptoms, were enrolled. Participants were included after providing informed consent in the Hindi language.

### 2.1. Sample size calculation

Our objective was to study fetomaternal outcome in women with or without PRES in preeclampsia/eclampsia with neurological symptoms and to study the frequency of occurrence of PRES in women with preeclampsia and eclampsia with neurological symptoms. With reference to a previous study by Zehra Kurdoglu et al.^14^ where he observed that gestational age at birth in PRES was 33.42 ± 4.74 (weeks) and in nonPRES was 33.50 ± 3.47 (weeks). Also, Stillbirth rate was 3.70%. This value was taken as reference, and the minimum required sample size with a margin of error 5.5% and a 5% level of significance is 46 patients.

## 3. Methodology

Women attending the Antenatal care (ANC) clinic at Safdarjung Hospital with singleton pregnancies beyond 20 weeks of gestation, diagnosed with preeclampsia or eclampsia and presenting with neurological symptoms, were enrolled in the study. Participants were included after meeting the inclusion criteria and providing informed consent in a language they understood, primarily Hindi. The informed consent was signed by the participants themselves and obtained in the gynecology emergency and labor room. All participants after stabilization underwent non-contrast computed tomography (NCCT) head or MRI brain during antenatal period or within 1 week postpartum. Findings of NCCT head were noted. In this study, all computed tomography (CT) scans were conducted post-delivery, after the patients were stabilized. This approach ensured that the fetus was not exposed to radiation during the prenatal period. Women with equivocal features on the NCCT head underwent MRI brain. Women with PRES features were followed up till delivery and discharge for fetomaternal outcomes. Frequency of occurrence of PRES was noted. Fetomaternal outcomes in women with PRES were compared to women without PRES.

All women underwent NCCT head using Seimens Somatom Definition AS 128, STRATON MX P model number 10414539. Women who had equivocal NCCT head finding underwent MRI brain using DISCOVERY MR 750 having system ID number 083027000011318. This is an observational study conducted in accordance with hospital protocols to prevent the development of PRES. Blood pressure was managed using injectable labetalol and oral nifedipine, with post-delivery administration of nitroglycerin (NTG). Seizures were promptly controlled with serum magnesium sulfate, following the Pritchard regime. Additionally, risk factors were identified early, allowing for timely and appropriate interventions. These measures were implemented to effectively mitigate the development of PRES in the study population.

Risk factors for PRES such as sepsis, shock, hemolytic uremic syndrome, acute glomerulonephritis, acute and chronic renal failure, parenchymal diseases, renal artery stenosis, immunosuppressive drugs, systemic lupus erythematosus, Sjogren’s disease, vasculitis, scleroderma, cryoglobulinemia, polyarteritis nodosa, Wegner’s granulomatosis, Bechet’s disease, Hashimoto’s thyroiditis, primary sclerosing cholangitis, thrombotic thrombocytopenic purpura, Henoch-schooling purpura, leukemia and lymphomas, sickle cell anemia, hemolytic uremic syndrome, acute porphyria, etc.

### 3.1. Outcome measures

The primary outcome of the study was to investigate fetomaternal outcomes in women with or without PRES in preeclampsia and eclampsia presenting with neurological symptoms. Maternal factors studied as outcomes included period of gestation, mode of delivery (normal vaginal delivery/instrumental delivery/cesarean section), abruptio placentae (abruption), cortical blindness, focal deficits, uncontrolled seizures, high dependency unit high dependency unit (HDU)/intensive care unit (ICU) stay, need for intubation, postpartum hemorrhage (PPH), stroke, and maternal mortality. Fetal factors studied as outcomes included stillbirth, prematurity, fetal growth restriction (FGR), meconium-stained liquor (MSL), APGAR score, respiratory distress syndrome (RDS), neonatal intensive care unit (NICU) stay, and early neonatal death (ENND). The secondary outcome was the frequency of women diagnosed with PRES in preeclampsia and eclampsia with neurological symptoms. In this study, imaging interpretations were subject to inter-reader variability, as multiple radiologists were involved in reviewing the CT and MRI scans. To address this, standardized radiological criteria were used for diagnosing PRES, ensuring consistency and reliability in the diagnosis across different readers.

## 4. Statistical Analysis

The data were entered into an MS Excel spreadsheet and analyzed using the statistical package for social sciences (SPSS) version 21.0. Categorical variables were presented as numbers and percentages (%), while continuous variables were presented as mean ± SD and median. The normality of the data was tested using the Kolmogorov-Smirnov test, and if rejected, a non-parametric test was employed.

### 4.1. Comparative analysis

For comparative analysis, categorical variables were compared between groups using the Chi-Square test or Fisher’s exact test, depending on the sample size and distribution. Continuous variables were compared using the Unpaired t-test for normally distributed data, and the Mann-Whitney Test was employed for data that did not meet normality assumptions. These tests allowed for the assessment of differences in fetomaternal outcomes between groups with and without PRES. The significance level for all statistical tests was set at a p-value of <0.05, indicating that differences with a *p*-value below this threshold were considered statistically significant.

## 5. Ethics

The Institutional Ethics Committee of the study center (Vardhman Mahavir Medical College and Safdarjung Hospital, New Delhi) reviewed the study protocol and informed consent form and approved the study (approved protocol dated 11.07.2022; approved no. IEC/VMCC/SIH/THESIS/06/2022/CC-166).

## 6. Results and Observations

In the present study, 59 out of 64 women (92.18%) were unscheduled, while 3 (4.68%) were registered and only 2 (3.12%) were booked.

In the study, 30 out of 64 women (46.8%) were between 26 and 30 years old, while 43.8% were between 19 and 25 years old, and only 9.4% were older than 30 years. Primigravida women were significantly more common in the PRES group compared to the non-PRES group (82.5% *vs*. 12.5%, *p* < 0.001). Therefore, the occurrence of PRES was higher in primigravida women compared to multigravida women in the present study. PRES was diagnosed using NCCT in 29 out of 40 women with PRES, resulting in a significantly higher detection rate (*p* < 0.001).

Out of 64 participants, 26 (40.6%) had severe preeclampsia, 21 (32.8%) had antepartum eclampsia, and 17 (26.6%) had postpartum eclampsia.

In the study, 55 women (85.9%) had headaches, 44 (68.8%) had blurred vision, 11 (17.2%) had uncontrolled seizures, 5 (7.8%) had strokes, 15 (23.4%) had focal deficits, and 2 (3.1%) had cortical blindness. The most common neurological symptoms observed were headaches and blurred vision, occurring in 85.9% and 68.8% of the women, respectively.

The fundus changes were observed in 90% of women with PRES. Fundus changes were significantly higher in PRES compared to nonPRES (*p* < 0.001).

In the study, 30 (75%) out of 40 women with PRES underwent cesarean section. It was observed that cesarean section rate was significantly higher in women with PRES (*p* < 0.001).

In the current study, PPH was observed in 40% of women with PRES compared to 4.2% in non-PRES cases. The PPH rate was significantly higher in women with PRES (*p* = 0.002). Abruption was noted in 27.5% of women with PRES and only 4.2% in those without PRES, indicating a significantly higher abruption rate in women with PRES (*p* = 0.023). Additionally, RDS occurred in 32 (80%) of women with PRES, contrasted with 2 (8.3%) in non-PRES cases, signifying a significantly elevated prevalence of RDS in women with PRES (*p* < 0.001).

## 7. Discussion

The present study was conducted in Department of Obstetrics and Gynecology, Department of Neurology, and Department of Radiodiagnosis at Vardhman Mahavir Medical College and Safdarjung Hospital, New Delhi. A total of 64 singleton pregnant women with a period of gestation >20 weeks with preeclampsia and eclampsia with neurological symptoms and fulfilling the eligibility criteria were recruited. All women after stabilization underwent NCCT head or MRI brain within 1 week of delivery to confirm diagnosis of PRES and were followed up for fetomaternal outcomes. Frequency of occurrence of PRES was noted, and also fetomaternal outcomes in women with PRES were compared to women without PRES.

## 8. Age (Years)

In the present study, the mean age of women with PRES was 21.45 years, and the age range was 18–26 years, and this was significantly lower than nonPRES (*p* < 0.001). Similar was seen in previous study where women with PRES were significantly younger (*p* = 0.005) with median age of 23 years and range of 20–30 years.^15^ Similarly, in an earlier study, PRES was observed in the age group of 20–25 years.^16^ Two past studies were in discordance to the present study with mean age 28 years and 32 years, respectively.^17,18^

## 9. Parity

In the present study, out of 64 participants, 56.2% of women were primigravida and 43.8% of women were multigravida, but the majority (82.5%) of women with PRES were primigravida and only 17.5% were multigravida. Past studies were also in concordance to the present study where PRES was seen primarily affecting primigravida at 81% and 59.1%, respectively.^16,17^ In contrast to the present study, in one previous study, PRES was seen higher in multigravida (57.6%),^15^ and in another study, PRES was found equally affecting both multigravida and primigravida.^18^

## 10. Period of Gestation (Weeks)

In the present study, the mean period of gestation of 64 participants was 35.36 weeks ranging from 28 to 40 weeks. The mean period of gestation in women with PRES was 34.02 weeks ranging from 28 to 37 weeks. Similarly, in previous studies, the babies were born preterm with mean period of gestation of 33 weeks and 36 weeks, respectively.^15,19^

## 11. Degree of Severity of HDP in Study Participants (Severe Preeclampsia/Antepartum Eclampsia/Postpartum Eclampsia)

In the present study, out of 64 participants, 26 (40.6%), 21 (32.8%), and 17 (26.6%) were of severe preeclampsia, antepartum eclampsia, and postpartum eclampsia, respectively. PRES was significantly higher (p = 0.005) in antepartum eclampsia (47.5%). This finding of present study was in concordance to the previous study where PRES was higher (80%) in antepartum eclampsia.^20^ It was in discordance to the past studies where PRES was reported more in women with postpartum eclampsia at 55% and 88%, respectively.^16,21^

### 11.1. Rationale behind respiratory distress syndrome (RDS) in women with PRES

The high incidence of RDS among women with PRES can be attributed to several interrelated factors. PRES is characterized by acute hypertension and end-organ damage, which can lead to significant systemic complications. The severe hypertension associated with PRES can result in increased vascular permeability and pulmonary edema, contributing to RDS.

Additionally, the presence of PRES often indicates a severe form of preeclampsia or eclampsia, conditions that themselves are known to increase the risk of pulmonary complications. In cases of severe preeclampsia, the body’s response to high blood pressure and associated systemic effects can compromise lung function, leading to the development of RDS.

The interplay between PRES and the physiological stress of severe preeclampsia may exacerbate respiratory distress, as both conditions place significant strain on the cardiovascular and respiratory systems. Furthermore, if PRES leads to other systemic complications, such as renal or cardiac dysfunction, these can further contribute to the development of RDS.

In summary, the occurrence of RDS in a significant proportion of women with PRES reflects the severe nature of the syndrome and its impact on overall organ function, particularly in the context of severe preeclampsia or eclampsia.

## 12. Headache

In the present study, all (100%) women with PRES experienced headache, signifying headache as a consistent symptom of cerebral involvement, which was similar to the previous studies where headache was reported in 50%, 58%, 68.57%, and 100%, respectively.^20,22–24^ This finding was in contrast with one past study where headache was reported more (89.5%) in nonPRES women.^15^

## 13. Focal Deficits

In the present study, focal deficits were reported in 23.4% of women with PRES which was in contrast lower to the past studies where it was observed in 40.82% and 51.1%, respectively.^17,21^ It was similar to previous studies where it was noted in 8.57% and 15.4%, respectively.^15,20^

## 14. Postpartum Hemorrhage (PPH)

In the current study, PPH was observed in 40% of women with PRES which was in contrary to past study where no PPH occurred.^16^

## 15. Abruption

In the present study, abruption was reported in 27.5% of women with PRES, and this was in discordance with previous study where no abruption was reported.^16^

## 16. Visual Disturbances (Blurring of Vision)

In the present study, blurring of vision was reported in 97.5% of women with PRES which was in contrast to the earlier studies where it was reported much lower, 33.33%, 33%, 34%, 28.57%, and 47.4%, respectively.^20,21,24,25,26^

It was similar to a previous study where it was reported in 71.43% of women with PRES.^17^

## 17. Fundoscopy

In the current study, in women with PRES grade 1, hypertensive retinopathy and papilledema were seen in 70% and 2.5%, respectively. Similar was observed in a past study where papilledema was seen in 4.1%.^22^ This was in contrary to a previous study where grade 1 hypertensive retinopathy and papilledema were found in 31.25% and 6.25%, respectively.^27^

## 18. Uncontrolled Seizures

In the present study, uncontrolled seizures were reported in 22.5% of women with PRES. This was in contrast to past studies where it was reported at much higher rate of 63.5%, 72%, and 62.5%, respectively.^22,23,25^

## 19. Cortical Blindness

In the present study, cortical blindness was observed in 5% of women with PRES. This was in contrast higher to the past studies where it was noted in 1.8% and 16.7%, respectively.^23,28^

## 20. Systolic Blood Pressure (SBP) (MM of HG)

In current study, mean systolic Blood Pressure (BP) was 167.10 mm of Hg and range 150–198 mm of Hg in women with PRES which was similar to earlier studies reporting mean systolic blood pressure of 162 mm of Hg, 160 mm of Hg, and 161 mm of Hg, respectively.^15,25,26,28^

## 21. Diastolic Blood Pressure (DBP) (MM of HG)

In the present study, mean diastolic BP in women with PRES was 106.67 mm of Hg. Similarly in previous studies diastolic BP were higher with mean of 100 mm of Hg, 102 mm of Hg, and 100 mm of Hg, respectively.^15,25,26^ This was in discordance to past study where it was 98 mm of Hg.^28^

## 22. AST (IU/L)

In the present study, mean value of aspartate aminotransferase (AST) was 298.95 IU/L (more than 7 times normal) in women with PRES which was in contrast to the past studies where mean values were 142, 47.15, 43, and 135 IU/L, respectively.^15,22,26,29^

## 23. ALT (IU/L)

In the present study, mean value of alanine transaminase (ALT) was 328.78 IU/L (8 times normal) in women with PRES which was in contrast to the previous studies where mean values were much lower having value of 253, 46.48, 29, and 101 IU/L, respectively.^15,22,26,29^

## 24. LDH Value (IU/L)

In the current study, mean LDH value was almost double (1008.05 IU/L) the normal value in women with PRES which was in discordance to previous studies with mean values of LDH of 453.03 and 553.5 IU/L, respectively.^26,29^

## 25. Mode of Delivery

In the current study, 75.0%, 22.5%, and 2.5% of women with PRES had cesarean section, vaginal delivery, and instrumental delivery (forceps-assisted vaginal delivery), respectively, and this finding was similar to past studies where cesarean section rate was high: 100%, 74% (antepartum eclampsia); 33% (postpartum eclampsia), 73%, 56.25%, and 89%, respectively.^15,21,25,27,28^

## 26. Fetal Growth Restriction (FGR)

In the current study, 77.5% of women with PRES had evidence of FGR which was similar to previous studies where FGR was reported in 83% and 55.6% of women, respectively.^18,28^

## 27. Stillbirth

In the current study, stillbirth was reported in 5.0% of women with PRES which was in concordance to past study where it was reported 6.7%^28^ and discordance to a previous study where much higher (25%) stillbirth rate was observed.^15^

## 28. APGAR Score (1 minute and 5 minutes)

In the present study, the mean 1 minute and 5 minutes APGAR score was 5.68 and 6.7, respectively, in women with PRES. The previous study was similar where the mean value of 1 minute and 5 minutes APGAR score was 4.76 and 6.47, respectively,^28^ and was in contrast to a past study where the much higher mean value of 5 minutes APGAR of 8 was observed.^18^

## 29. Neonatal Intensive Care Unit (NICU) Stay

In the current study, NICU stay was observed in 34% of women with PRES which was similar to the past study with 46.6% of NICU stay.^28^

## 30. Early Neonatal Death (ENND)

In the current study, ENND was observed in 20% of women with PRES which was similar to past study with 19% ENND.^28^

## 31. Maternal Mortality

In the current study, 6 (15%) maternal mortalities were reported in women with PRES and 1 (4.16%) mortality among nonPRES. This was in concordance to past study where 6 (17.14%) maternal mortalities were reported.^20^ This was in contrast to previous studies where no maternal mortalities occurred ^21,27^ and other past studies where maternal mortalities of 2 (6%) and 12 (11.5%) were reported, respectively.^22,25^

## 32. Frequency of PRES

In the current study, 40 (62.5%) out of 64 women were diagnosed as PRES either on NCCT or MRI. Similar was found in previous studies where frequency of occurrence of women with PRES was 55.5%, 77.14%, 62.3%, 88.8%, and 83.3%, respectively.^18,23,26–28^ In contrast, many past studies had reported much lower occurrences of PRES in 22.6%, 32.07%, and 45.9% of women, respectively.^15,26,29,30^

### 32.1. Overall pathophysiology

PRES results from severe hypertension that disrupts endothelial cell function, causing increased permeability of blood vessels. This disruption leads to the leakage of fluid into surrounding tissues, including the lungs, contributing to pulmonary edema. In the case of severe preeclampsia or eclampsia, which often accompanies PRES, the elevated blood pressure and systemic inflammation further exacerbate this condition. The accumulation of fluid in the alveoli impairs normal gas exchange, leading to RDS.

The systemic effects of PRES and severe preeclampsia, such as compromised cardiovascular function and increased fluid overload, contribute to the development of RDS. This high prevalence of RDS reflects the severe impact of PRES on overall organ function and the body’s ability to manage fluid balance effectively.

### 32.2. Strengths

The study employed a prospective cohort design, recruiting women with severe preeclampsia and eclampsia presenting with neurological symptoms to diagnose PRES and observe fetomaternal outcomes.

### 32.3. Limitation

A notable limitation of this study was its small sample size, limiting its generalizability to larger populations. Future research would benefit from larger study populations to enhance the robustness of findings.

## 33. Conclusion

The study found that women with PRES experienced significantly worse fetomaternal outcomes, including longer ICU stays, higher rates of intubation, abruption, PPH, caesarean sections, FGR, prematurity, RDS, and MSL. PRES was more common in women with antepartum eclampsia, first pregnancies, and younger ages. Neurological symptoms such as headaches, blurred vision, and focal deficits were prevalent, with cortical blindness being a potential indicator of PRES. NCCT was effective for diagnosing PRES when MRI was unavailable. Early identification of PRES in women with severe preeclampsia and neurological symptoms can improve management and ensure timely referral.

## Abbreviations

**Table:** 

ACOG	American College of Obstetricians and Gynaecologists
ADC	Apparent Diffusion Coefficient
ANC	Antenatal care
AOL	Augmentation of Labour
APGAR	Appearance, Pulse, Grimace, Activity, and Respiration
AST/ALT	Aspartate Aminotransferase/Alanine Transaminase
BP	Blood Pressure
CNS	Central Nervous System
DIC	Disseminated Intravascular Coagulation
DWI	Diffusion Weighted Imaging
ECHO	Echocardiogram
ECG	Electrocardiogram
EEG	Electroencephalogram
ENND	Early Neonatal Death
FGR	Fetal Growth Restriction
FLAIR	Fluid Attenuated Inversion Recovery
GCS	Glasgow Coma Scale
GTCS	Generalized Tonic Clonic Seizures
HDP	Hypertensive Disorder of Pregnancy
HELLP	Hemolysis Elevated Liver Enzymes and Low Platelet Count
HDU	High Dependency Unit
ICH	Intracranial Hemorrhage
ICU	Intensive Care Unit
IOL	Induction of Labour
LDH	Lactate Dehydrogenase
LSCS	Lower Segment Cesarean Section
mRS	Modified Rankin Score
MRI	Magnetic Resonance Imaging
MRA	Magnetic Resonance Angiography
MRP	Magnetic Resonance Pancreatography
MSL	Meconium-Stained Liquor
NCCT	Non-contrast Computed Tomography
NER	National Eclampsia Registry
POG	Period of Gestation
PPH	Postpartum Hemorrhage
PRES	Posterior Reversible Encephalopathy Syndrome
RDS	Respiratory Distress Syndrome
RPLS	Reversible Posterior Leukoencephalopathy Syndrome
SWAN	Susceptibility Weighted Angiography
SPECT	Single Photon Emission Computed Tomography
Tc99m	Technetium 99

## Definitions

**Unscheduled:** “Unscheduled” refers to patients who have not attended any prenatal visits or appointments prior to their hospital admission.

**Preeclampsia:** Preeclampsia is a pregnancy complication characterized by new-onset hypertension (blood pressure ≥140/90 mm Hg) and proteinuria (≥300 mg in a 24-hour urine collection or a protein/creatinine ratio ≥0.3) occurring after 20 weeks of gestation. It can also present with other symptoms such as edema and elevated liver enzymes, but these are not required for diagnosis.

**Severe Preeclampsia:** Severe preeclampsia is a more serious form of preeclampsia that involves significantly elevated blood pressure (≥160/110 mm Hg) and/or severe proteinuria (≥5 g in a 24-hour urine collection). It may also include additional complications such as severe headaches, visual disturbances, epigastric pain, or signs of end-organ dysfunction (e.g., elevated liver enzymes or thrombocytopenia). This condition necessitates more intensive monitoring and management to prevent progression to eclampsia and other severe outcomes.

The examination was conducted by ophthalmologists, and multiple ophthalmologists were involved in the evaluation.

## Approval by the Human Research Ethics Committee

Obtaining ethical clearance from the Institutional Ethical Committee (IEC/VMMC/SJH/Thesis/06/2022/CC-166).

## Acknowledgment

I appreciate the encouragement from my friends, colleagues, and family. Lastly, I thank all the patients who participated in this study.

## Conflict of Interest Statement

The authors declare no conflicts of interest.

## Author Contribution Statement

SN: Conceptualization, Data curation, Formal analysis, Investigation, Methodology. US: Conceptualization, Data curation, Formal analysis, Investigation, Methodology, Project administration, Resources, Supervision, Validation. YA: Resources, Supervision, Validation. AL: Resources, Supervision, Validation. SC: Resources, Supervision, Validation. SP: Resources, Software, Validation.

## Figures and Tables

**Figure 1.**
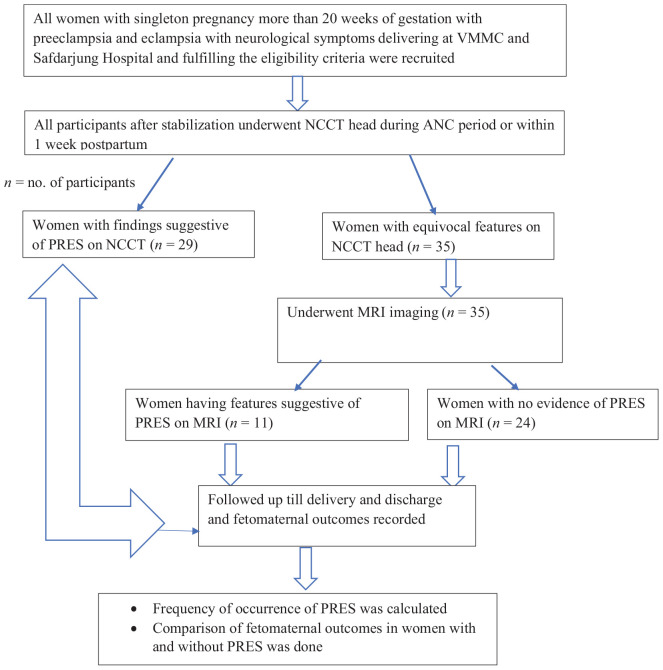
Study flow chart.

**Table 1.** Demographic profile.

**Distribution of the participants in terms of different age groups (years) (*n* = 64)**					
**Age**	**Number**	**Percentage**		**95% CI**	
19–25 years	28	43.8%		31.6–56.7%	
26–30 years	30	46.8%		34.5–59.7%	
>30 years	6	9.4%		3.9–19.9%	
**Association between PRES and parity**					
**Parity**	**PRES**			**Chi-squared test**	
	**Yes**	**No**	**Total**	**χ^2^**	***P* value**
Primigravida	33 (82.5%)	3 (12.5%)	36 (56.2%)	29.867	< 0.001
Multigravida	7 (17.5%)	21 (87.5%)	28 (43.8%)		
**Association between PRES and NCCT findings**					
Yes	29 (72.5%)	0 (0.0%)	29 (45.3%)	31.817	< 0.001
No	11 (27.5%)	24 (100.0%)	35 (54.7%)		

**Table 2.** Distribution in terms of degree of severity of HDP (hypertensive disorder of pregnancy) (*n* = 64).

**Degree of severity of HDP**	**Number**	**Percentage**	**95% CI**
Severe preeclampsia	26	40.6%	28.8–53.6%
Antepartum eclampsia	21	32.8%	21.9–45.8%
Postpartum eclampsia	17	26.6%	16.7–39.3%

**Table 3.** Distribution in terms of neurological symptoms: headache, blurring of vision, cortical blindness, uncontrolled seizures, stroke, focal deficits (*n* = 64).

**Symptoms**	**Symptoms: headache**	**Number**	**Percentage**	**95% CI**
Headache	Yes	55	85.9%	74.5–93.0%
	No	9	14.1%	7.0–25.5%
Focal deficits	Yes	15	23.4%	14.1–36.0%
	No	49	76.6%	64.0–85.9%
Uncontrolled seizures	Yes	11	17.2%	9.3–29.1%
	No	53	82.8%	70.9–90.7%
Stroke	Yes	5	7.8%	2.9–18.0%
	No	59	92.2%	82.0–97.1%
Cortical blindness	Yes	2	3.1%	0.5–11.8%
	No	62	96.9%	88.2–99.5%
Blurring of vision	Yes	44	68.8%	55.8–79.4%
	No	20	31.2%	20.6–44.2%

**Table 4.** Association between PRES and fundus examination (*n* = 64).

	**PRES**			**Fisher’s exact test**	
**Fundus examination**	**Yes**	**No**	**Total**	**χ^2^**	***P* value**
None	4 (10.0%)	22 (91.7%)	26 (40.6%)	41.595	< 0.001
Grade 1 hypertensive retinopathy	28 (70.0%)	2 (8.3%)	30 (46.9%)		
Grade 2 hypertensive retinopathy	5 (12.5%)	0 (0.0%)	5 (7.8%)		
Grade 3 hypertensive retinopathy	2 (5.0%)	0 (0.0%)	2 (3.1%)		
Papilledema	1 (2.5%)	0 (0.0%)	1 (1.6%)		

**Table 5.** Association between PRES and mode of delivery (*n* = 64).

	**PRES**			**Fisher’s exact test**	
**Mode of delivery**	**Yes**	**No**	**Total**	**χ^2^**	***P* value**
Normal vaginal delivery	9 (22.5%)	19 (79.2%)	28 (43.8%)	19.657	< 0.001
Cesarean section	30 (75.0%)	5 (20.8%)	35 (54.7%)		
Instrumental delivery	1 (2.5%)	0 (0.0%)	1 (1.6%)		

**Table 6.** Complications specifically related to postpartum hemorrhage (PPH), abruption, and respiratory distress syndrome (RDS) (*n* = 64).

**Association between PRES and postpartum hemorrhage (PPH)**					
	**PRES**			**Chi-squared test**	
**Postpartum hemorrhage**	**Yes**	**No**	**Total**	**χ^2^**	***P* value**
Yes	16 (40.0%)	1 (4.2%)	17 (26.6%)	9.874	0.002
No	24 (60.0%)	23 (95.8%)	47 (73.4%)		
**Association between PRES and abruption**					
Yes	11 (27.5%)	1 (4.2%)	12 (18.8%)	5.361	0.023
No	29 (72.5%)	23 (95.8%)	52 (81.2%)		
**Association between PRES and respiratory distress syndrome (RDS) in babies**					
Yes	32 (80.0%)	2 (8.3%)	34 (53.1%)	30.938	< 0.001
No	8 (20.0%)	22 (91.7%)	30 (46.9%)		
